# Assessment of alterations in histone modification function and guidance for death risk prediction in cervical cancer patients

**DOI:** 10.3389/fgene.2022.1013571

**Published:** 2022-09-19

**Authors:** Tingting Zhao, Bairong Liu, Mengyuan Zhang, Shiguo Li, Can Zhao, Li Cheng

**Affiliations:** ^1^ Department of Obstetrics and Gynecology, The Seventh Affiliated Hospital, Sun Yat-sen University, Shenzhen, Guangdong, China; ^2^ Information Department, The Seventh Affiliated Hospital, Sun Yat-sen University, Shenzhen, Guangdong, China; ^3^ Medical Administration Division, The Seventh Affiliated Hospital, Sun Yat-sen University, Shenzhen, Guangdong, China

**Keywords:** cervical cancer, histone modification, prognosis, signature, prediction

## Abstract

**Background:** Cervical cancer is the second most lethal malignancy among women, and histone modification plays a fundamental role in most biological processes, but the prognostic value of histone modification in cervical cancer has not been evaluated.

**Methods:** A total of 594 cervical cancer patients from TCGA-CESC, GSE44001, and GSE52903 cohorts were enrolled in the current study, along with the corresponding clinicopathological features. Patients with a follow-up time less than one month were removed. A total of 122 histone modification-associated signaling pathways were obtained from the MSigDB. The activation scores of these pathways were evaluated using the “GSVA” package, differentially expressed genes were identified by the “limma” package, and pathway enrichment was conducted using the “clusterProfiler 4.0” package. The subsequent least absolute shrinkage and selection operator (LASSO) regression analysis was performed using the “glmnet” package, and a prognostic nomogram was established using the “regplot” package. For the prediction of potential therapeutic drugs, we used the data from GDSC2016 and visualized them via “MOVICS”.

**Results:** Nine of 23 histone modification-associated prognostic genes were identified to construct the prognostic signature by LASSO analysis, named the histone modification-associated gene (HMAG) signature. Cervical patients with HMAG-H in TCGA-CESC cohort showed a 2.68-fold change of death risk, with the 95% CI from 1.533 to 4.671 (*p* < 0.001), as well as the increased death risk of HMAG-H in the GSE44001 cohort (HR: 2.83, 95% CI: 1.370–5.849, *p* = 0.005) and GSE44001 cohort (HR: 4.59, 95% CI: 1.658–12.697, *p* = 0.003). We observed the preferable AUC values of the HMAG signature in TCGA-CESC cohort (1-year: 0.719, 3-year: 0.741, and 5-year: 0.731) and GSE44001 cohort (1-year: 0.850, 3-year: 0.781, and 5-year: 0.755). The C-index of the nomogram showed a prognostic value as high as 0.890, while the C-index for age was only 0.562, and that for grade was only 0.542. Patients with high HMAG scores were more suitable for the treatment of CHIR-99021, embelin, FTI-277, JNK-9L, JQ12, midostaurin, PF-562271, pyrimethamine, and thapsigargin, and patients with low HMAG scores were more suitable for the treatment of BMS-536924, CP466722, crizotinib, PHA-665752, rapamycin, and TAE684.

**Conclusion:** We comprehensively evaluated the histone modification status in cervical cancer patients and revealed histone modification-associated prognostic genes to construct the HMAG signature, aiming to provide a new insight into prognosis prediction and precise clinical treatment.

## Introduction

Currently, cervical carcinoma has become the second most lethal malignancy among women worldwide (Bray et al., 2018), with 527,624 new cases and 265,672 tumor-specific deaths annually (Shrestha et al., 2018). For the accurate prediction of prognosis, the International Federation of Gynecology and Obstetrics put forward the cervical cancer staging standard in 2018, according to the depth, greatest dimension of stromal invasion, and the extension of tumor on adjacent regions (such as vagina and pelvis), separating cervical cancer into stages I, II, III, and IV and further substages (Balcacer et al., 2019; Salvo et al., 2020). For different stages of the tumor, the treatments are diverse. Surgical interventions, including trachelectomy and radical hysterectomy, are performed in most early cervical cancers. In addition, radiation and chemoradiation are applied in most advanced or metastatic cervical cancers (Johnson et al., 2019). Unfortunately, these treatments still result in a low response rate and poor prognosis. Therefore, it is essential to develop new predictive prognostic models for optimizing treatment strategies.

Histone proteins, a type of abundant cellular protein, are surrounded by DNA to make nucleosomes (Taylor et al., 2020). The N-terminal tail of each histone protein is the site of posttranslational modifications (PTMs) and can make contact with adjacent nucleosomes (Bannister and Kouzarides, 2011; Bartke et al., 2013; Taylor et al., 2020). There are numerous types of histone PTMS, including acetylation, methylation, ubiquitinoylation, and phosphorylation (Bannister and Kouzarides, 2011; Taylor et al., 2020; Zhang et al., 2021). They play fundamental roles in most biological processes (Bannister and Kouzarides, 2011; Taylor et al., 2020). Histone methylation functions in many levels of transcriptional regulation from the chromatin architecture to specific locus regulation and RNA processing (Greer and Shi, 2012). Histone acetylation influences myriad cellular and physiological processes, including transcription, phase separation, autophagy, mitosis, differentiation, and neural function (Shvedunova and Akhtar, 2022). Histone ubiquitinoylation works in organization of repair to preserve genomic integrity after the breaking of the DNA double strand (Uckelmann and Sixma, 2017). Histone phosphorylation provides a rapid and reversible physiological response to DNA damage, nutritional stress, or an altered metabolic state (Uckelmann and Sixma, 2017). More importantly, histone modification affects the accessibility of DNA and recruitment of DNA-binding proteins and thereby regulates gene transcription, which controls transcriptional regulation and corresponding disorders. For example, modification of histones by acetylation/deacetylation influences gene expression and is therefore related to the carcinogenic process (Audia and Campbell, 2016) (Fang et al., 2014).

Many molecular mechanisms of histone modification have also been found. A high level of histone acetylation is associated with the expression of proto-oncogenes; nonetheless, a low level of histone acetylation is linked with the silencing of tumor suppressor genes (Armenta-Castro et al., 2020). Moreover, it has been reported that the repressive expression of osteoprotegerin (OPG) genes is associated with histone modification and further causes chromatin architecture alterations and results in transcriptional repression of OPG genes. OPG plays a tumor suppressive role in tumorigenesis; therefore, histone modification-associated OPG suppression can accelerate the deterioration of cervical cancer (Lu et al., 2009). Some studies also suggest that histone acetylation is linked with the expression of the LGALS9 gene. Histone acetylation is an activator modification for the LGALS9 gene, which can be observed in active promoters. This modification can promote LGALS9 gene transcription. Galectin-9, which is encoded by the LGALS9 gene, can have a positive effect on the apoptosis of tumor cells. Histone acetylation of LGALS9 reduces deterioration in cervical cancer (Armenta-Castro et al., 2020). Relevant molecular mechanisms have been studied to some extent. However, the impact of changes in signaling pathways caused by histone modification on the progression of cervical cancer is not clear. Accordingly, we carried out the current study to illuminate the prognostic value and function of histone modification in cervical cancer patients.

In this study, we gathered information on the signaling pathways related to histone modification. According to the activation status of the pathways, we identified cervical cancer patients with activated and suppressed histone modification. Then, we sought differentially expressed genes (DEGs) to reflect the inner alteration of the tumor caused by histone modification. Among these DEGs, we screened for relevant genes that best reflected prognosis and constructed a histone modification-associated gene (HMAG) signature to predict the prognosis of patients with cervical cancer.

## Methods

### Data collection

We obtained the gene expression profile and corresponding clinical information of CESC patients from three independent clinical cohorts: TCGA-CESC, GSE44001, and GSE52903. We downloaded the TCGA-CESC dataset from the GDC TCGA project via the R package “TCGAbiolinks.” The transcripts per million (TPM) format of the gene expression file was chosen and then transformed to the log2 (TPM+1) type to make it comparable with the sequencing results from the microarray. Patient samples in the GSE52903 cohort were collected from Mexico City, which were HPV16-positive and fresh samples with more than 70% tumor tissues. The clinical outcome recorded in TCGA-CESC and GSE52903 was the overall survival (OS). Samples in the GSE44001 dataset were collected from South Korea, and gene expression values were detected using the GPL14951 Illumina HumanHT-12 WG-DASL V4.0 R2 expression beadchip platform, along with the prognosis information of disease-free survival (DFS). We further filtered the enrolled patients to exclude those without paired gene expression data and clinical information and those with a follow-up time of less than one month to decrease the potential bias (Table 1).

**TABLE 1:** Clinicopathological information of the enrolled cohorts.

	TCGA-CESC (N = 248)	GSE44001 (N = 295)	GSE52903 (N = 51)	Overall (N = 594)
Survival time				
Mean (SD)	29.0 (35.5)	49.3 (24.5)	44.0 (26.5)	40.4 (31.2)
Median [Min, Max]	16.5 [1.02, 195]	48.8 [3.50, 104]	58.0 [1.00, 86.0]	36.4 [1.00, 195]
Events^a^				
No	189 (76.2%)	258 (87.5%)	31 (60.8%)	478 (80.5%)
Yes	59 (23.8%)	37 (12.5%)	20 (39.2%)	116 (19.5%)
Age				
Mean (SD)	47.9 (13.9)	-	50.9 (14.4)	48.4 (14.0)
Median [Min, Max]	46.0 [20.0, 80.0]	-	50.0 [24.0, 74.0]	46.0 [20.0, 80.0]
Missing	0 (0%)	295 (100%)	0 (0%)	295 (49.7%)
Stage				
Stage I	134 (54.0%)	254 (86.1%)	24 (47.1%)	412 (69.4%)
Stage II	56 (22.6%)	41 (13.9%)	8 (15.7%)	105 (17.7%)
Stage III	34 (13.7%)	0 (0%)	15 (29.4%)	49 (8.2%)
Stage IV	19 (7.7%)	0 (0%)	4 (7.8%)	23 (3.9%)
Unknown	5 (2.0%)	0 (0%)	0 (0%)	5 (0.8%)
Grade				
Unknown	26 (10.5%)	295 (100%)	51 (100%)	372 (62.6%)
G1	15 (6.0%)	0 (0%)	0 (0%)	15 (2.5%)
G2	111 (44.8%)	0 (0%)	0 (0%)	111 (18.7%)
G3	95 (38.3%)	0 (0%)	0 (0%)	95 (16.0%)
G4	1 (0.4%)	0 (0%)	0 (0%)	1 (0.2%)

a The clinical outcome recorded in TCGA-CESC and GSE52903 cohorts is overall survival (OS), and in the GSE44001 cohort, it is disease-free survival (DFS).

### Removal of batch effects between cohorts

For the three enrolled cohorts, the potential nonbiological bias was eliminated to make the gene expression profiles of different cohorts more comparable. The “sva” R package was applied with its ComBat algorithms to remove the batch effects, and the gene expression profiles were all adjusted. Then, GSE44001 and GSE52903 were combined as the GEO-combined cohort. Three cohorts were detached before the removal of the batch effect (SupplementartyFigure S1A) and fused together after removal (SupplementartyFigure S1B).

### Collection of the histone modification pathways

To comprehensively reveal the diverse distribution of histone modification in cervical cancer patients, we collected a total of 122 histone modification-associated signaling pathways from the Molecular Signatures Database (MSigDB)-C5 (Liberzon et al., 2011): ontology gene sets, including the process of histone-mediated phosphorylation, methylation, ubiquitination, and acetylation.

### Gene set variation analysis

The activation of 122 histone modification-associated signaling pathways was assessed by the “GSVA, v.3.5” R package, the enrichment score for a specific gene set in each sample was calculated, and GSVA quantified the total gene set activation results (Hanzelmann et al., 2013). Therefore, the gene expression profiles were transferred to gene set activation profiles, including the activated scores of 122 signaling pathways for each cohort.

### Revealing the differentially expressed genes and pathways

To identify the diverse downstream altered biological processes, we carried out the differentially expressed genes (DEGs) among histone modification-activated and -suppressed subgroups via the “limma” package, with the preset threshold value of *p* < 0.05 and |log2fc| > 0.4. Pathway enrichment was performed by the “clusterProfiler 4.0” R package (Wu et al., 2021), with the employment of 50 HALLMARK pathways and KEGG pathways. In addition, Metascape (http://metascape.org) (Zhou et al., 2019) was also used to annotate the DEGs to reveal the activated pathways.

### Construction and calculation of the HMAG signature

The prognostic value of the aforementioned identified DEGs was further assessed by univariate Cox regression analysis. Gene expression was first separated into high and low groups and then entered into the univariate Cox regression analysis. The prognostic genes in both the TCGA-CESC cohort and GEO-combined cohort were selected for a subsequent least absolute shrinkage and selection operator (LASSO) regression analysis, which was conducted using the “glmnet” package. The selected genes were used to calculate the risk score by adding the gene expression multiplied by the corresponding coefficient and named the HMAG score. The HMAG signature was trained in the TCGA-CESC cohort and validated in the GSE44001 and GSE52903 cohorts. The HMAG score was calculated for each patient and then applied for the subsequent analysis.

### Multivariate analysis and establishment of the nomogram

The prognostic value of the HMAG score in each cohort was assessed by the K-M plot and receiver operating characteristic (ROC) curve. Multivariate Cox regression analysis was also performed to adjust the potential impact of other clinical features and is presented with a forest plot. Furthermore, we employed the “regplot” package to establish the clinical prognostic nomogram, which could provide a quantitative method for the individualized prediction of cervical cancer. Factors that emerged from the multivariate Cox regression analysis were enrolled for the establishment of the nomogram. C-index curve, calibration curve, and decision curve analyses were all performed to validate the clinical usefulness and accuracy of the nomogram via the “rms” and “rmda” packages.

### Revealing activated pathways and potential therapeutic drugs

We assessed the activated pathways by fast gene set enrichment analysis (fgsea). First, GSEA is performed with the ranking of input molecular readouts, and then the pathway enrichment score is calculated by walking down the list of features, which means that if a feature is felled into the target pathway, the running-sum statistic increases; otherwise, it decreases. The final score is the maximum deviation from zero encountered in the random walk and normalized by computing the z-score of the estimate compared to a null distribution obtained from a random permutation. Moreover, the highly expressed genes in high-HMAG and low-HMAG groups were also annotated to reveal the activated biological process by clusterProfiler 4.0 and visualized via the treeplot model.

For the prediction of potential therapeutic drugs, we used the data from GDSC2016 (https://www.cancerrxgene.org/) and visualized them via “MOVICS” (Lu et al., 2020). Ridge regression analysis was performed to predict the potential response result to the chemotherapy drug of each patient and represented by the estimated inhibitory concentration (IC_50_); the lower the IC_50_ was, the higher the effectiveness of the drug treatment was (Geeleher et al., 2014). Moreover, we also searched for potential therapeutic new drugs through the Gene Set Cancer Analysis (GSCA) online website (http://bioinfo.life.hust.edu.cn/GSCA).

### Comparison of the HMAG signature with published signatures

To assess the prognostic value of the HMAG signature carried out in the current study, we searched published articles to collect signatures from other studies. Finally, we used four signatures, namely, immune-associated genes (Yu et al., 2021), DNA damage repair-associated genes (Zhou et al., 2022), ferroptosis-related genes (Qi et al., 2021), and autophagy-related genes (Chen et al., 2020). The risk score of each signature was calculated in the TCGA-CESC cohort, and the ROC value and C-index were used to evaluate the prognostic value.

### Statistics

The log-rank test was used to compare the survival outcome between two groups, Student’s *t*-test was used to compare the distribution between two groups, and Fisher’s exact test was performed to distinguish the difference in categorical data. All statistical analyses were performed using R (Version: 4.1.2). A two-tailed *p*-value < 0.05 was recognized as statistically significant.

## Results

### Characterizing the diversified histone modification-activated status and altered signaling pathways

**FIGURE 1:**
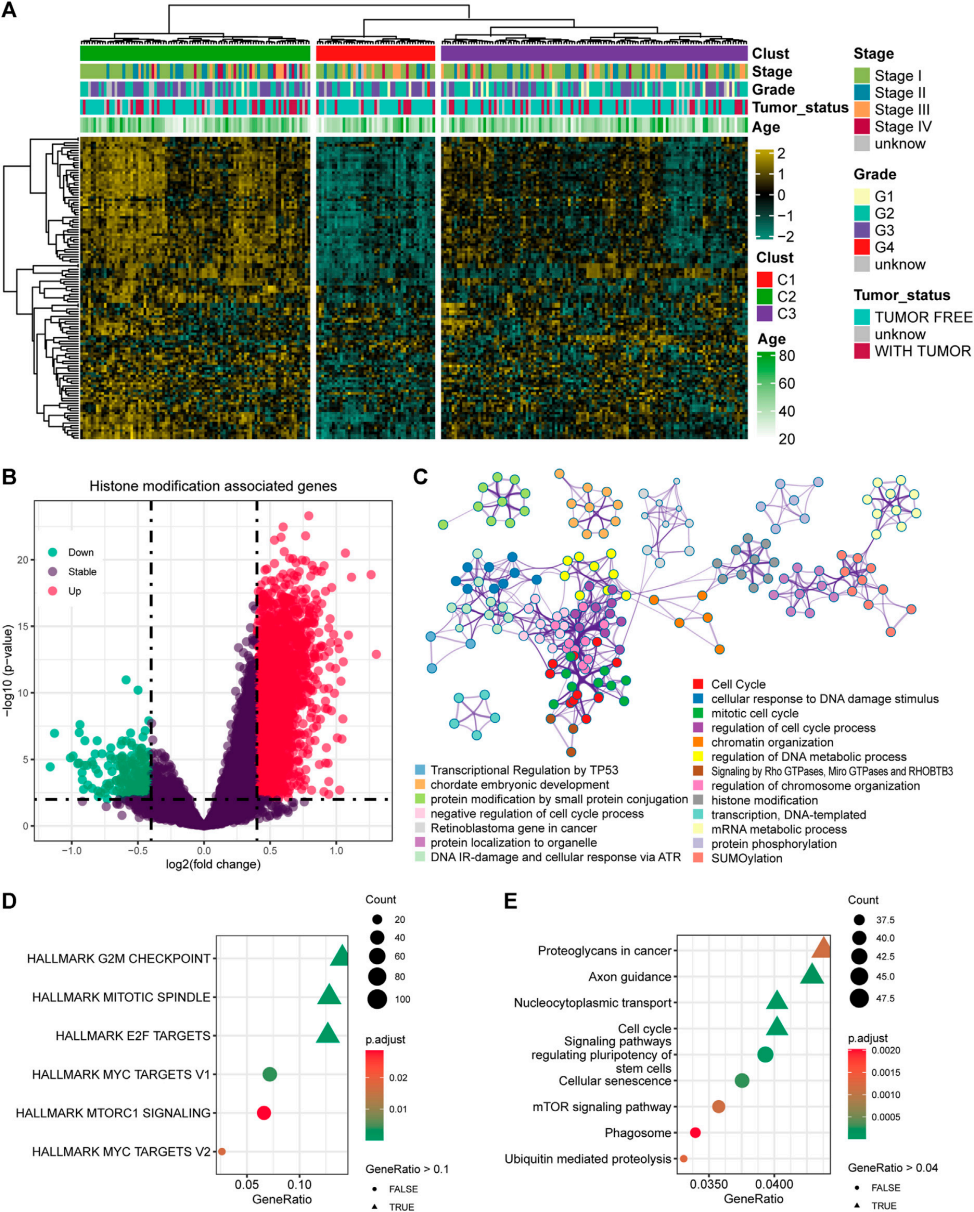
Identification of the histone modification-altered genes and pathways. **(A)** Heatmap showing the activation status and clinical features in cervical cancer patients. **(B)** Differentially expressed genes (DEGs) between the histone modification-activated and -suppressed subgroups. **(C)** Enriched signaling pathways of the DEGs using Metascape. **(D)** Enriched signaling pathways of the DEGs by HALLMARK pathways. **(E)** Enriched signaling pathways of the DEGs by KEGG pathways.

A total of 122 histone modification-associated pathways were obtained from MSigDB-C5, and the activated score was generated based on the GSVA quantification. Then, the 248 cervical cancer patients from TCGA-CESC cohort were divided into three subtypes based on the distanceMatrix function of the “ClassDiscovery” package, with the preset parameters of “euclidean” and “ward.D.” We observed diversified histone modification activation in C1, C2, and C3. The patients in C2 contained the most activated status of histone modification, while the C1 patients contained the suppressed histone modification (Figure 1A). Then, we compared the DEGs between C2 and C1 (Figure 1B), with a preset threshold value of *p* < 0.05 and |log2fc| > 0.4. A total of 2040 genes were upregulated in C2, and the other 200 genes were upregulated in C1. We combined the DEGs between C1 and C2, annotated the enriched signaling pathways, and observed the altered biological processes of the cell cycle, cellular response to DNA damage stimulus, chromatin organization, and DNA metabolic process (Figure 1C). In addition, we also validated the DEGs in the HALLMARK and KEGG pathways. The DEGs targeted the G2/M checkpoint, mitotic spindle, and E2F targets (Figure 1D) and were also linked with proteoglycans in cancer, axon guidance, nucleocytoplasmic transport, and cell cycle pathways in KEGG pathways (Figure 1E).

### Distinguishing the prognostic genes and constructing the signature

**FIGURE 2:**
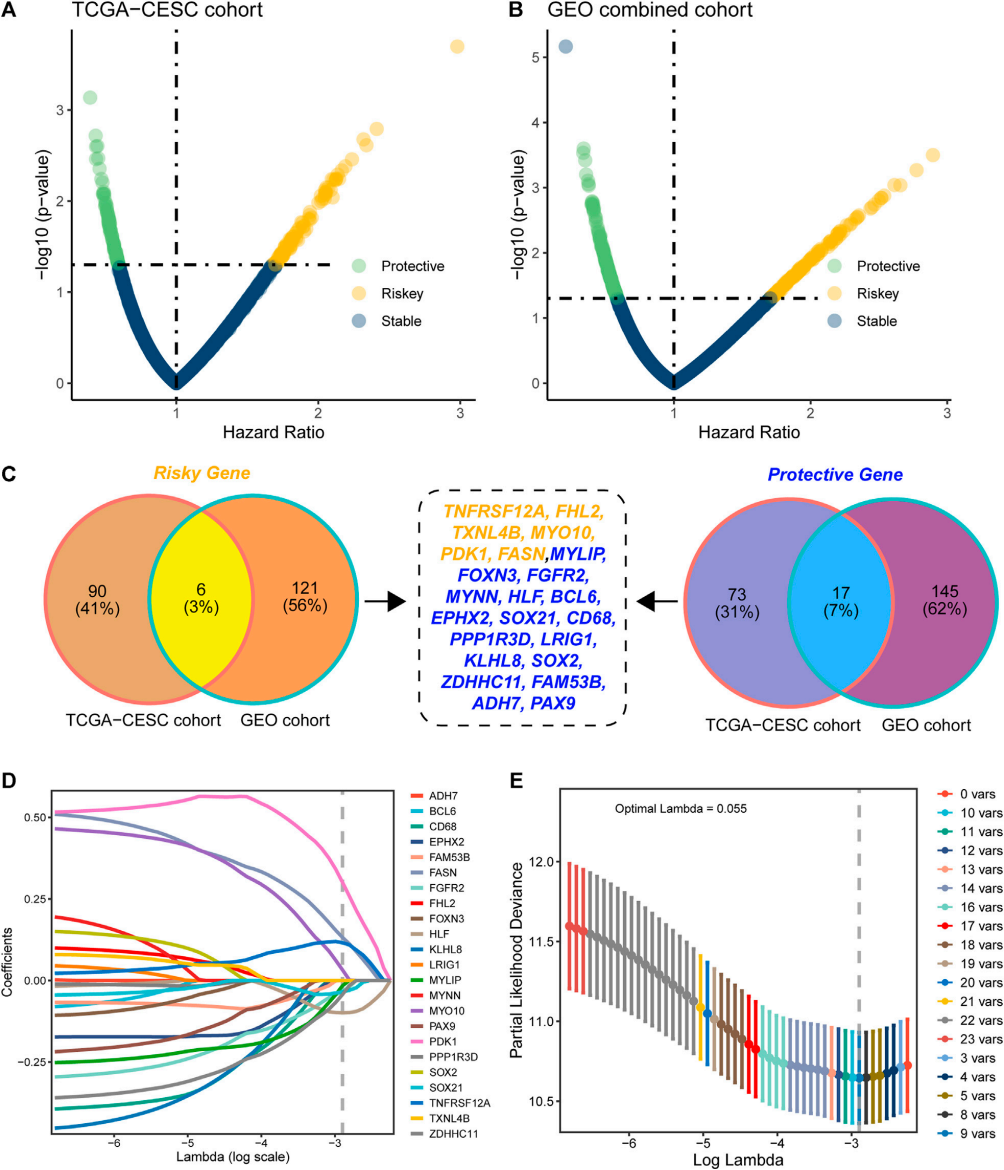
Construction of the histone modification-associated gene signature. **(A)** Selection of the prognostic genes in TCGA-CESC cohort; **(B)** selection of the prognostic genes in the GEO-combined cohort; **(C)** Venn plot showing the risk genes and protective genes in both TCGA-CESC and GEO-combined cohorts; **(D)** optimal tuning parameter (lambda) in the LASSO analysis selected with 10-fold cross-validation and one standard error rule; and **(E)** LASSO coefficient profiles of the 23 candidate genes.

Based on the 2240 DEGs, we assessed and selected the prognostic DEGs in TCGA-CESC cohort (Figure 2A) and GEO-combined cohort (Figure 2B). Genes with an HR higher than 1 and a *p*-value less than 0.05 were regarded as risky genes, while genes with an HR less than 1 and a *p*-value less than 0.05 were regarded as protective genes. Ninety-six risk genes from TCGA-CESC cohort and 127 risk genes from the GEO-combined cohort merged out six common risk genes; 90 protective genes from TCGA-CESC cohort and 162 protective genes from the GEO-combined cohort merged out 17 common protective genes (Figure 2C). Therefore, 23 genes were finally enrolled for LASSO analysis, and nine genes were identified under the best optimal lambda value of 0.055, leading to the prognostic genes SOC21, HLF, FGFR2, MYLIP, ZDHHC11, FASN, PDK1, MYO10, and TNFRSF12A (Figures 2D,E). The HMAG signature was calculated using the following formula: HMAG score = 0.115713151 * TNFRSF12A + 0.032286676 * MYO10 + 0.300717617 * PDK1 + 0.131853347 * FASN + -0.015900246 * MYLIP - 0.033923872 * FGFR2 - 0.099329649 * HLF - 0.034241118 * SOX21 - 0.042765486 * ZDHHC11.

### Prognostic effectiveness of the HMAG signature

**FIGURE 3:**
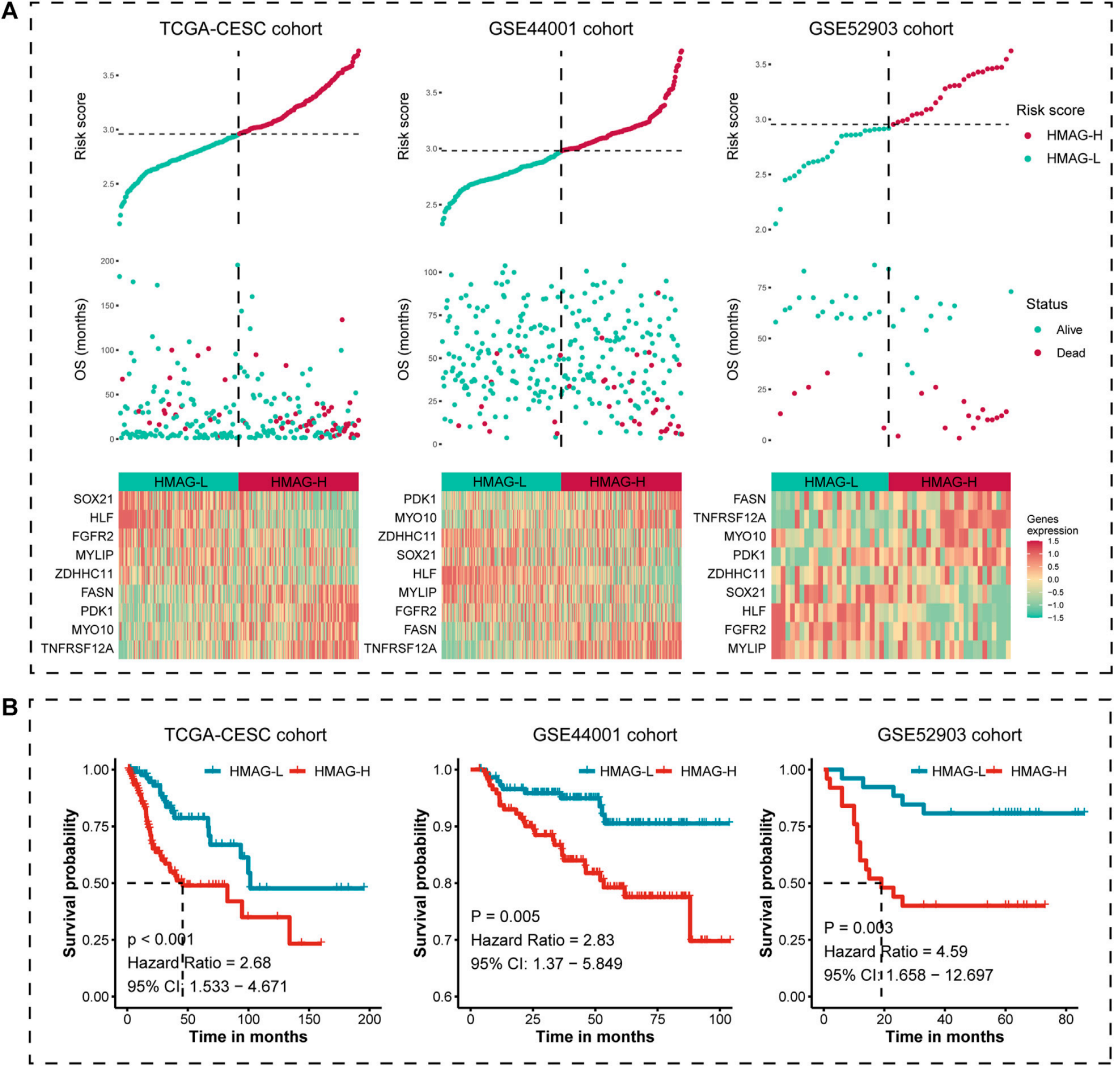
Risk map and K-M plot for TCGA-CESC, GSE44001, and GSE52903 cohorts. **(A)** Risk score distribution (upper), clinical outcome distribution (middle), and gene expression distribution (bottom) for TCGA-CESC, GSE44001, and GSE52903 cohorts. **(B)** K-M plot of the HMAG-L score and HMAG-H score subgroups for TCGA-CESC, GSE44001, and GSE52903 cohorts.

We calculated the HMAG score of each patient based on the aforementioned formula. The overall distributions of the risk score, survival status, and gene expression profiles of the nine-gene signature in TCGA-CESC cohort (left), the GSE44001 cohort (middle), and the GSE52903 cohort (right) are shown in Figure 3A. Patients in each cohort were separated into HMAG-L and HMAG-H based on the median HMAG score, and the prognostic value was assessed by Cox regression analysis. Cervical patients with HMAG-H in TCGA-CESC cohort showed a 2.68-fold change of death risk, with the 95% CI from 1.533 to 4.671 (*p* < 0.001), as well as the increased death risk of HMAG-H in the GSE44001 cohort (HR: 2.83, 95% CI: 1.370–5.849, *p* = 0.005) and GSE44001 cohort (HR: 4.59, 95% CI: 1.658–12.697, *p* = 0.003) (Figure 3B).

**FIGURE 4:**
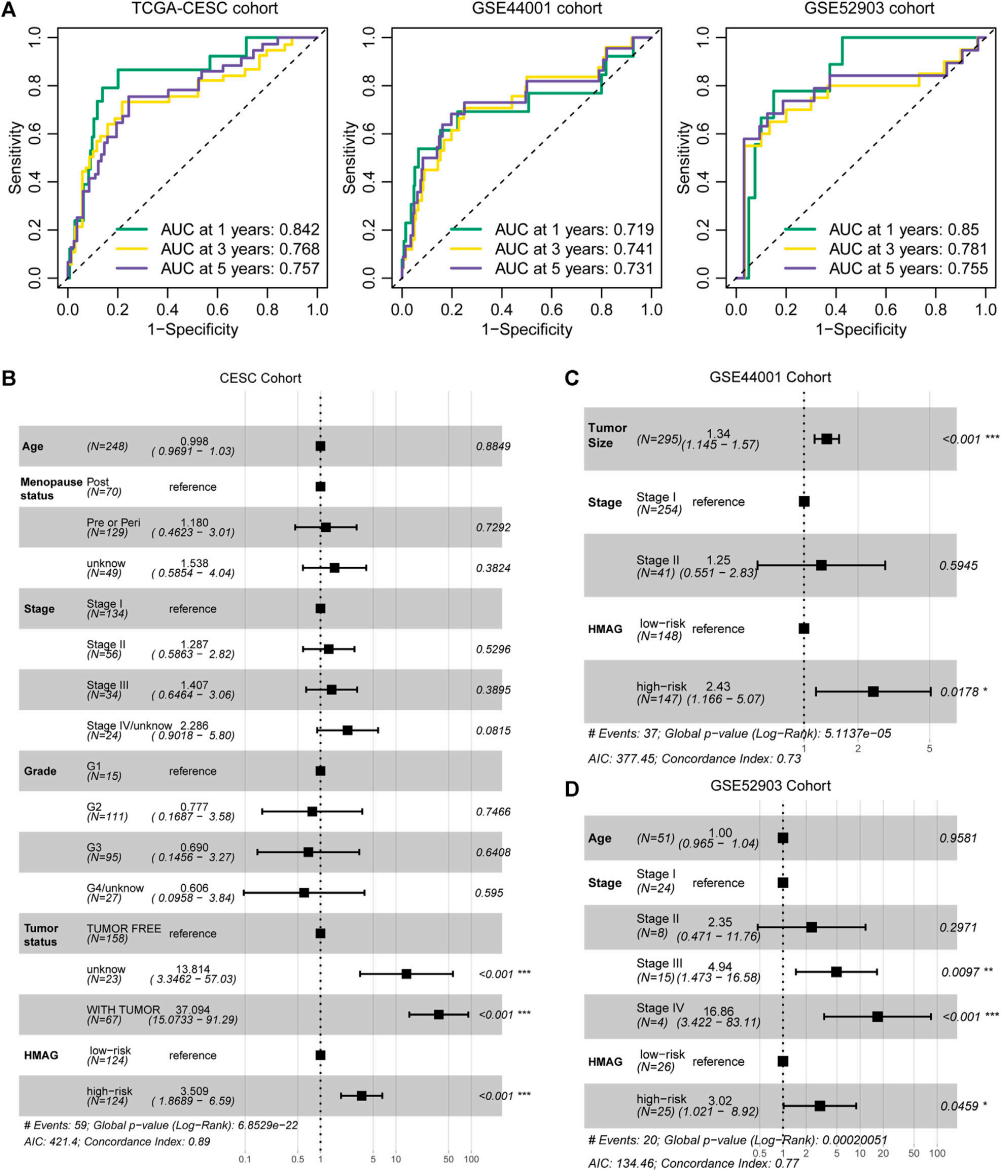
Evaluation of the prognostic value of the HMAG signature. **(A)** 1-year, 3-year, and 5-year AUC values in TCGA-CESC, GSE44001, and GSE52903 cohorts; **(B)** forest plot showing the prognostic value of the HMAG signature after adjusting for clinical features in TCGA-CESC cohort; **(C)** forest plot showing the prognostic value of the HMAG signature after adjusting for clinical features in the GSE44001 cohort; and **(D)** forest plot showing the prognostic value of the HMAG signature after adjusting for clinical features in the GSE52903 cohort.

The prognostic accuracy was evaluated by ROC curves. We observed the preferable AUC values of the HMAG signature in TCGA-CESC cohort (1-year: 0.719, 3-year: 0.741, and 5-year: 0.731) and GSE44001 cohort (1-year: 0.850, 3-year: 0.781, and 5-year: 0.755) (Figure 4A). In addition, we also conducted multivariate Cox regression analysis to adjust for the impact of clinicopathological features. We observed that the HMAG signature (HR: 3.509, 95% CI: 1.869–6.590, *p* < 0.001) and tumor status (tumor vs. tumor free: HR: 37.094, 95% CI: 15.073–91.290, *p* < 0.001) were independent prognostic factors for cervical cancer patients in TCGA-CESC cohort but age, menopausal status, tumor stage, and tumor grade were not (Figure 4B). In the GSE44001 cohort, the HMAG signature also showed an independent risk factor after adjusting for tumor size and tumor stage (HR: 2.43, 95% CI: 1.166–5.070, *p* = 0.0178, Figure 4C). Similar results were also shown in the GSE52903 cohort (HR: 3.02, 95% CI: 1.021–8.920, *p* = 0.0459, Figure 4D).

### Prognostic nomogram established by the HMAG score and tumor status

**FIGURE 5:**
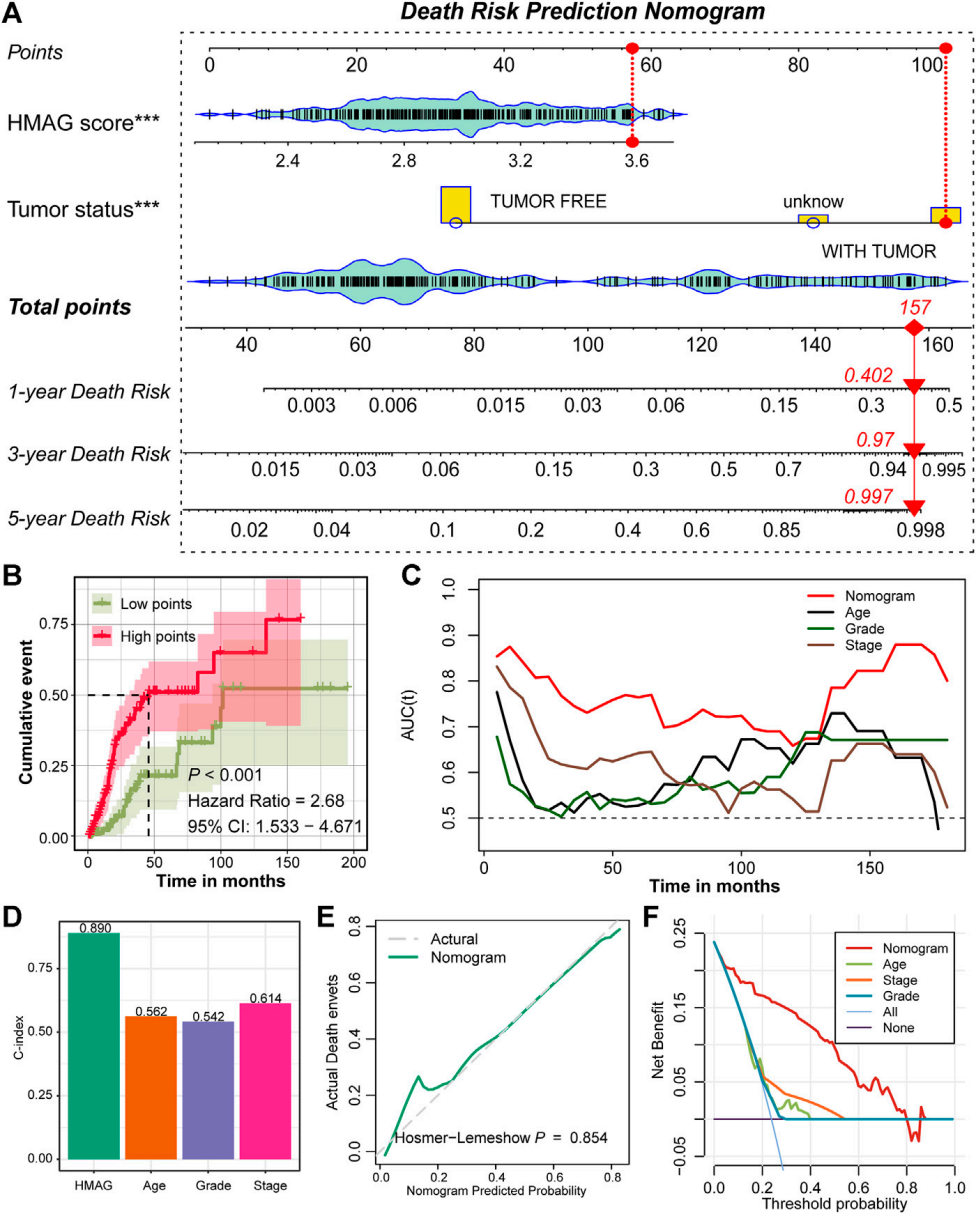
Prognostic nomogram constructed by the HMAG signature and tumor status. **(A)** Establishment of a nomogram combining the tumor status and HMAG signature; **(B)** K-M plot showing the diverse cumulative death event in low points and high points subgroups; **(C)** time ROC lines showing the prognostic value of nomogram, age, grade, and tumor stage; **(D)** bar plots showing the C-index of nomogram, age, grade, and tumor stage; **(E)** calibration plot for the nomogram. The dashed line represents the ideal nomogram, the solid line represents our nomogram, and a *p*-value of 0.854 indicates that the nomogram-predicted probability is very close to the actual death events; **(F)** DCA showed that our nomogram had the greatest net benefit compared with the single factor of age, stage, or grade.

With the aforementioned results, we established a prognostic nomogram model containing the factors HMAG score and tumor status (Figure 5A). For a specific patient, the HMAG score and tumor status correspond to the point, and the summary of two points is the total point, with a straight line from the total point site to the bottom line of 1-year, 3-year, and 5-year death risks, indicating the risk of death. With a cumulative incidence plot, we visualized the estimated probability of the death event prior to a specified time. The patients were separated into a low-point subgroup and a high-point subgroup, and we observed significantly diverse cumulative events in the two groups (HR: 2.68, 95% CI: 1.533–4.671, *p* < 0.001, Figure 5B). The AUC values changed over the follow-up time and presented a better prognostic value of the nomogram than regardless of age, grade, or stage (Figure 5C). The overall C-index of the nomogram showed a prognostic value as high as 0.890, while the C-index for age was only 0.562, for grade was only 0.542, and for stage was only 0.614 (Figure 5D). A *p*-value of 0.854 calculated by the Hosmer‒Lemeshow analysis in the calibration plot indicated that the prediction performance of this nomogram might be equivalent to an ideal predictive model (Figure 5E). DCA was performed to demonstrate a high clinical net benefit that was almost over the entire threshold probability of the nomogram model compared with other features (Figure 5F).

### Revealing activated pathways and potential therapeutic drugs

**FIGURE 6:**
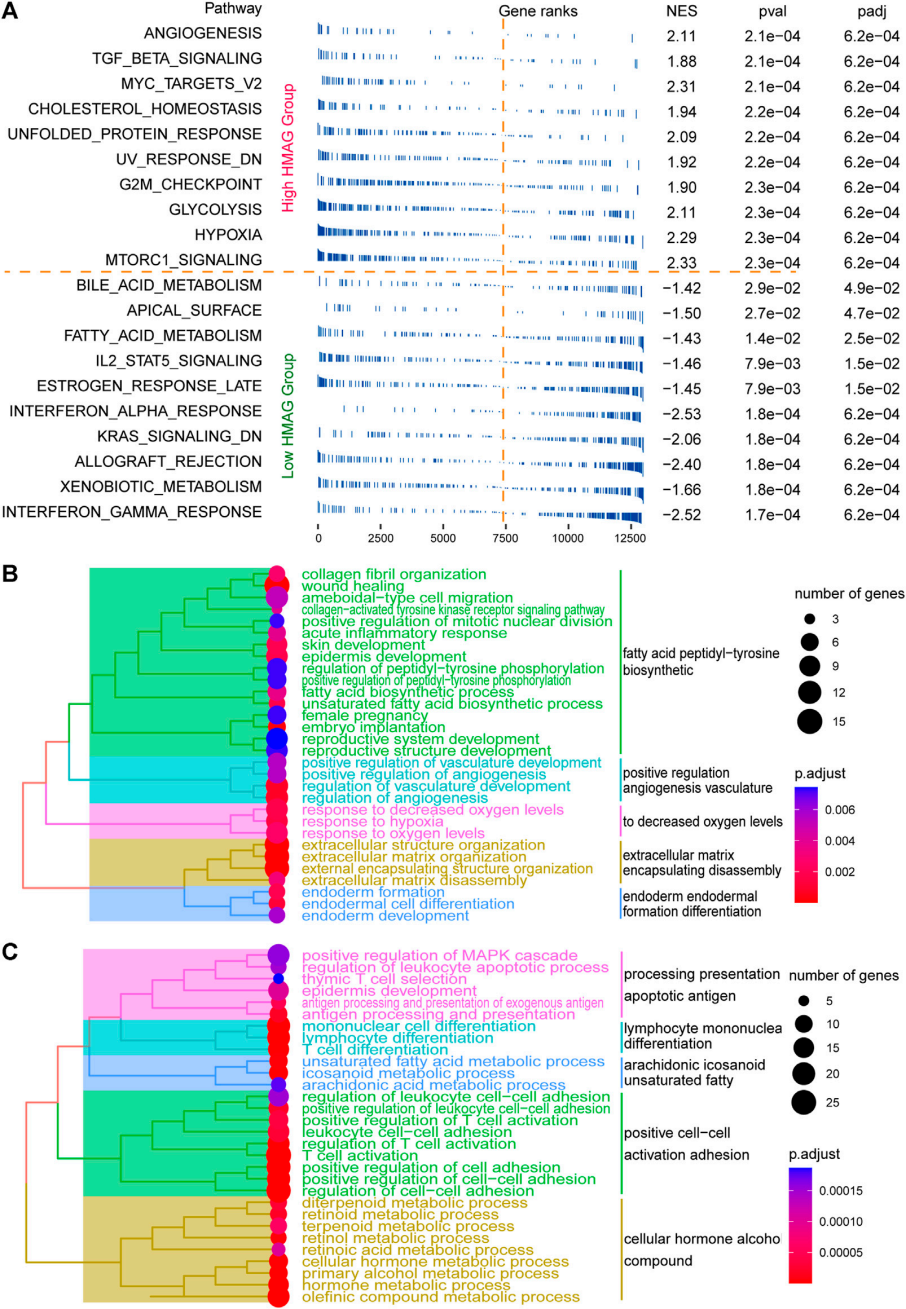
Revealing HMAG signature-regulated biological pathways. **(A)** fgsea showing the diverse activation of tumor biological pathways in the high-HMAG-score group and the low-HMAG-score group; **(B)** hierarchical clustering of enriched terms in HMAG upregulated genes; and **(C)** hierarchical clustering of enriched terms in HMAG downregulated genes.

The fgsea analysis was performed on TCGA-CESC cohort based on the tumor HALLMARK pathways. We revealed that patients with high HMAG scores contained the activated tumor pathways of angiogenesis, G2/M checkpoint, MYC targets, and hypoxia, while patients with low HMAG scores contained the activated tumor pathways of interferon gamma response, interferon alpha response, and IL2/STAT5 signaling (Figure 6A). The DEGs between the high and low HMAG score subgroups also indicated that the pathways of fatty acid peptidyl-tyrosine biosynthetic, positive regulation angiogenesis vasculature, decreased oxygen levels, extracellular matrix encapsulating disassembly, and endoderm formation differentiation terms were activated in high HMAG score patients (Figure 6B), and the pathways of apoptotic antigen presentation processing, lymphocyte mononuclear differentiation, arachidonic eicosanoid unsaturated fatty, positive cell‒cell activation adhesion, and cellular hormone alcohol compound terms were activated in low HMAG score patients (Figure 6C).

**FIGURE 7:**
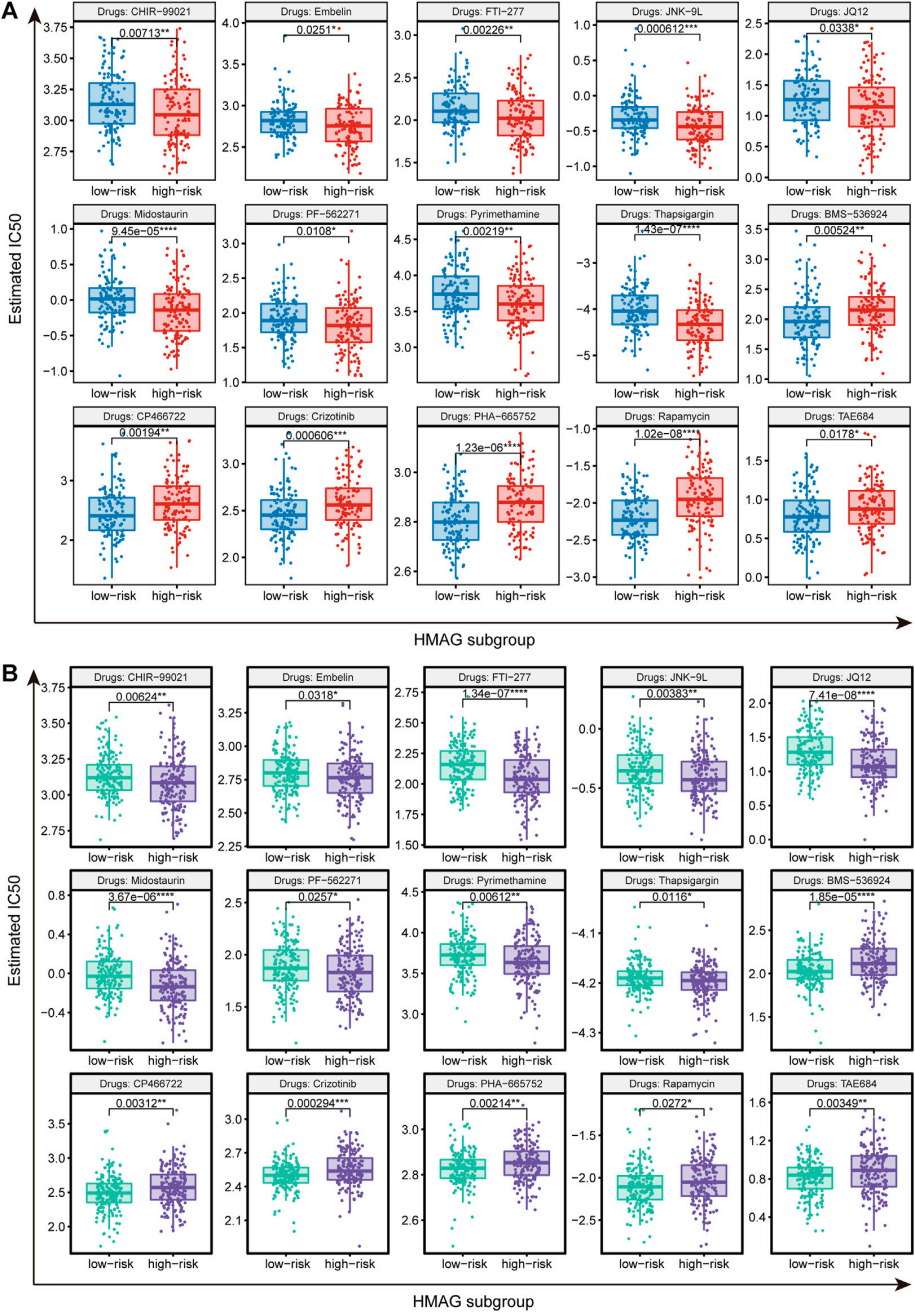
Prediction of therapeutic drugs for the HMAG low- and high-risk groups. **(A)** Potential therapeutic chemo drugs identified in TCGA-CESC cohort and **(B)** potential therapeutic chemo drugs validated in the GEO cohort.

We identified suitable therapeutic drugs based on the GDSC2016 database and revealed that patients with high HMAG scores were more suitable for the treatment of CHIR-99021, embelin, FTI-277, JNK-9L, JQ12, midostaurin, PF-562271, pyrimethamine, and thapsigargin, and patients with low HMAG scores were more suitable for the treatment of BMS-536924, CP466722, crizotinib, PHA-665752, rapamycin, and TAE684 (all *p* < 0.05, Figure 7A). Similar results were also validated in the GEO-combined cohort (all *p* < 0.05, Figure 7B). We also searched for new drugs from the GSCA dataset to treat the specific alteration of a single gene, and a total of 41 components were identified that might be functional in the clinical treatment of cervical cancer patients (Table 2).

**TABLE 2:** Predicting potential chemo drugs *via* the Cancer Therapeutics Response Portal dataset.

	FASN				
Drug	Dinaciclib	Alvocidib	PF-3758309	Tivantinib	Fluorouracil
Correlation	−0.20608	-0.1595	-0.15792	-0.14442	-0.13778
FDR	0.000186	0.005129	0.004505	0.010745	0.000318
	PDK1				
Drug	Triazolothiadiazine	Vincristine	Merck60	Leptomycin B	CR-1-31B
Correlation	−0.27131	−0.26454	−0.26955	−0.24879	−0.24931
FDR	5E-14	1.23E-13	1.66E-13	5.49E-12	7.46E-12
	MYO10				
Drug	Abiraterone	BRD-K99006945	VAF-347	ML334 diastereomer	PD318088
Correlation	-0.29579	-0.20423	-0.18466	-0.14235	-0.12552
FDR	0.01593	0.00329	0.001264	0.025005	0.003259
	TNFRSF12A				
Drug	Dasatinib	VAF-347	FGIN-1-27	BRD-K17060750	ML334 diastereomer
Correlation	−0.23802	−0.2234	−0.22125	−0.18099	−0.1755
FDR	1.67E-09	9.87E-05	0.047048	9.99E-05	0.006256
	SOX21				
Drug	Cytochalasin B	Simvastatin	Fluvastatin		
Correlation	0.107668	0.106844	0.104478		
FDR	0.022199	0.042931	0.027822		
	HLF				
Drug	Trametinib	Dasatinib	Selumetinib	GDC-0879	PD318088
Correlation	0.153191	0.137379	0.135254	0.121013	0.102907
FDR	0.022601	0.001002	0.001815	0.009436	0.018969
	FGFR2				
Drug	Tigecycline	Dabrafenib	Teniposide	Isoliquiritigenin	KW-2449
Correlation	0.250424	0.24845	0.2464	0.241401	0.238217
FDR	9.66E-06	0.000105	2.15E-06	0.003093	1.02E-10
	MYLIP				
Drug	ML334 diastereomer	Simvastatin	Lovastatin		
Correlation	0.155813	0.135817	0.122877		
FDR	0.014317	0.008074	0.005918		
	ZDHHC11				
Drug	BRD-K99006945	AT7867	ABT-199	Fluvastatin	AZD4547
Correlation	0.148094	0.1128	0.107107	0.102471	0.100118
FDR	0.032198	0.005013	0.049336	0.031639	0.025494

### The HMAG signature showed better prognostic value than other published signatures

**FIGURE 8:**
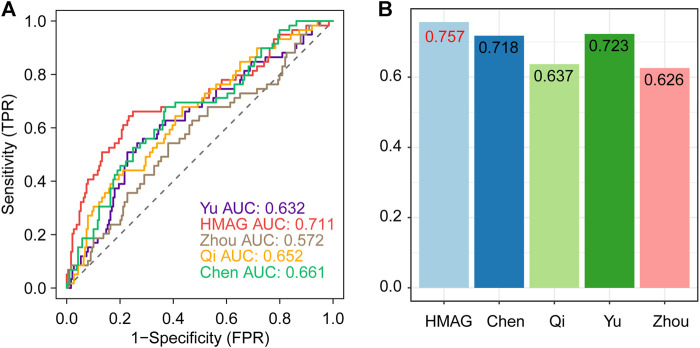
Comparison of the prognostic value between HMAG and proposed signatures. **(A)** ROC curves showing the prognostic value of five signatures and **(B)** C-index showing the prognostic value of five signatures.

We collected the published signatures of the genes and corresponding indexes from four studies, referring to the genes in immunity, DNA damage repair, ferroptosis, and autophagy. The risk score of each signature was calculated along with the HMAG score using the formula. We observed that the HMAG signature showed more excellent prognostic value than any other signature based on the AUC value (HMAG: 0.711, Yu et al.: 0.632, Zhou et al.: 0.572, Qi et al.: 0.652, and Chen et al.: 0.661, Figure 8A) and C-index (HMAG: 0.757, Yu et al.: 0.723, Zhou et al.: 0.626, Qi et al.: 0.637, and Chen et al.: 0.718, Figure 8B).

## Discussion

Epigenetic modifications are reversible and do not change the DNA sequence of genetic material but can alter how our body reads the DNA sequence, including the basic forms of DNA methylation, posttranslational modifications on histones and noncoding RNAs. However, regulation of the epigenome can distinctly lead to gene malfunction, regulating cell differentiation, proliferation, and even apoptosis and causing disordered cell growth and tumorigenesis. The abnormal control of posttranslational modification-related enzymes, including histone methylase, demethylase, acetylase, and acetyltransferase, acts as a pivotal risk factor for tumors, and these epigenetic alterations may lead to the reprogramming of genomes, activation of oncogenes, or silencing of tumor suppressors (Berry and Janknecht, 2013). Epigenetic regulators can impact the level of histone modification to the enhancer activity of genes via histone methyltransferases or acetylases (Xiao et al., 2022). Histone deacetylases (HDACs) are essential for maintaining the balance of cell processes by altering histone deacetylation. The abnormal expression of HDASs is tightly linked with several cancers, and their inhibitors have been applied in the clinic to treat several cancers (Patel et al., 2022). Moreover, histone modification impacts the process of epithelial–mesenchymal transition (EMT), and histone acetylation can modulate the acetylation levels of distinct histones at the promoters of EMT-related markers, EMT-inducing transcription factors, and EMT-related long noncoding RNAs to control EMT (Kong et al., 2022).

Histone modification function also emerges in the carcinogenesis of cervical cancer. Higher staining of H3K9ac indicates low grading, negative N-status, and low T-status in cervical cancer. Moreover, the increased expression of H3K4me3 in the cytoplasm was observed to be associated with advanced T stage and unfavorable prognosis in cervical cancer patients (Beyer et al., 2017). Galectin-9, which is encoded by LGALS9, is evidently detected in normal epithelium and endocervical glands but not in cervical intraepithelial neoplasia and cervical squamous cell carcinoma, which indicates that decreased Galectin-9 is a biomarker for the malignant potential of cervical cancer (Liang et al., 2008). Armenta-Castro et al. (2020) reported that histone H3K9 and H3K14 acetylation at the promoter of LGALS9 genes is tightly associated with the protein level of Galectin-9, and the LGALS9 gene presented higher levels of histone acetylation in normal cervical cells than in cancer cells. Sun et al. (2022) also reported that the inhibition of HDACs can activate mitophagy by mediating Parkin acetylation and leading to the inhibition of cervical cancer cell proliferation. Valproic acid/sodium valproate (VPA), a well-known antiepileptic agent, inhibits histone deacetylases, induces histone hyperacetylation, promotes DNA demethylation, and affects the histone methylation status in some cell models. Rocha et al. demonstrated that VPA promotes the abundance of H3K4me2/me3 and increases methyltransferase KMT2D gene expression in HeLa cells. Meanwhile, VPA can also induce the hypomethylation of H3K9me2 and concomitant with the increased gene expression of KDM3A (Rocha et al., 2022). Based on the aforementioned evidence, we sensed the important role of histone modification in cervical cancer tumorigenesis; therefore, we evaluated the histone modification pathway activation status in cervical cancer and generated a prognostic model to predict clinical prognosis.

In the current study, we enrolled a total of 594 cervical cancer patients. We first evaluated the histone modification activation status of 248 patients from TCGA-CESC cohort and identified the activated and suppressed subgroups. Further gene expression variation analysis filtered out 2240 genes and merged them with the results of prognostic analysis. Twenty-three histone modification-associated prognostic genes were enrolled in the LASSO Cox regression analysis. Eventually, we constructed the HMAG signature to predict the prognosis for cervical cancer patients, with nine genes: TNFRSF12A, MYO10, PDK1, FASN, MYLIP, FGFR2, HLF, SOX21, and ZDHHC11. The HMAG signature showed a preferable prognostic value in TCGA-CESC, GSE44001, and GSE52903 cohorts, and the HMAG signature is an independent prognostic factor after adjusting for the influence of other clinicopathological features. In addition, we established a prognostic nomogram combining the HMAG score and tumor status. The nomogram showed better prognostic value than did age, tumor grade, or tumor stage, with a C-index as high as 0.890. Currently, precise treatment raises concerns in the clinic, and we predicted suitable chemotherapy drugs for patients with high or low HMAG scores. The index of SOX21 in the HMAG signature formula is 0.034241118, which means that the higher expression of SOX21 is associated with a favorable prognosis. The elevated expression of histone H3R26-methylase CARM1 can increase the level of Sox21, and CARM1 inhibition can decrease Sox21 levels, which highlights the importance of epigenetic regulation of Sox21 (Goolam et al., 2016). Caglayan et al. (2013) demonstrated that the exogenous expression of Sox21 in tumor cells resulted in a significant decrease in the tumor size. It seems that Sxo21 appears to inhibit stem-like cell properties and initiate the aberrant differentiation of glioma cells. Kurtsdotter et al. also reported that the high levels of SOX5/6/21 in human primary glioblastoma cells enabled the expression of CDK inhibitors and decreased p53 protein turnover, which blocked their tumorigenic capacity through cellular senescence and apoptosis. FASN is a multienzyme protein that serves as the key regulator in lipid metabolism, especially fatty acid synthesis. KDM5C is a histone H3K4-specific demethylase, and the overexpression of KDM5C led to the reduction of H3K4me3 on the promoter and the corresponding downregulation of FASN expression to inhibit FASN-mediated lipid metabolism (Zhang et al., 2020). Du et al. (2022) illustrated that FASN can promote the lymph node metastasis of cervical cancer via cholesterol reprogramming and lymph angiogenesis. The mechanisms of how histone modification influences the tumorigenesis of cervical cancer are complicated and need a further in-depth study.

## Conclusion

In summary, we comprehensively evaluated the histone modification status in cervical cancer patients and revealed histone modification-associated prognostic genes to construct the HMAG signature, aiming to provide new insights into prognosis prediction and precise clinical treatment.

## Data Availability

The original contributions presented in the study are included in the article/Supplementary Material; further inquiries can be directed to the corresponding authors.

## Supplementary material

Supplementary Figure S1 Data pretreatment of TCGA-CESC, GSE44001 and GSE52903 cohorts.
