# Supplementary figures and images for: Assessment of alterations in histone modification function and guidance for death risk prediction in cervical cancer patients
Source: Front Genet. 2022 Sep 19;13:1013571. doi: 10.3389/fgene.2022.1013571 (PMC9527294; doi:10.3389/fgene.2022.1013571)

A

Raw PCA for combined expression profile

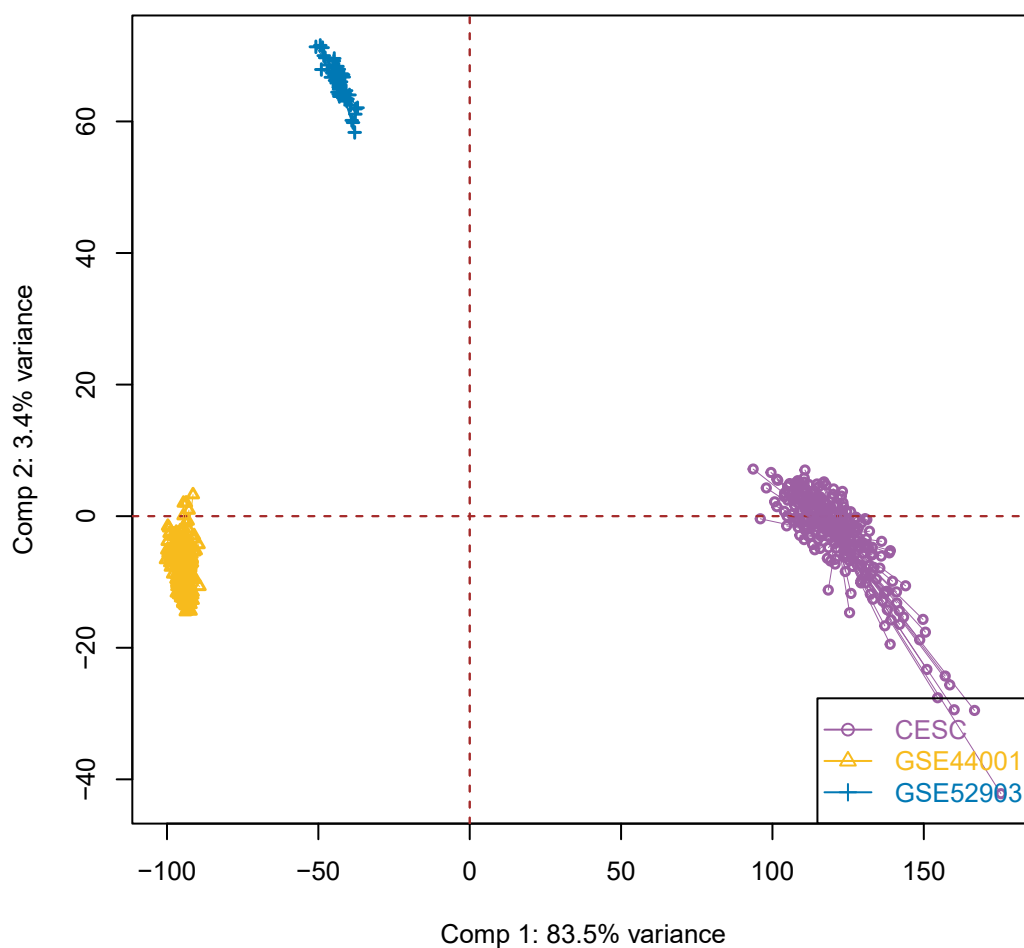

B

Combat PCA for combined expression profile

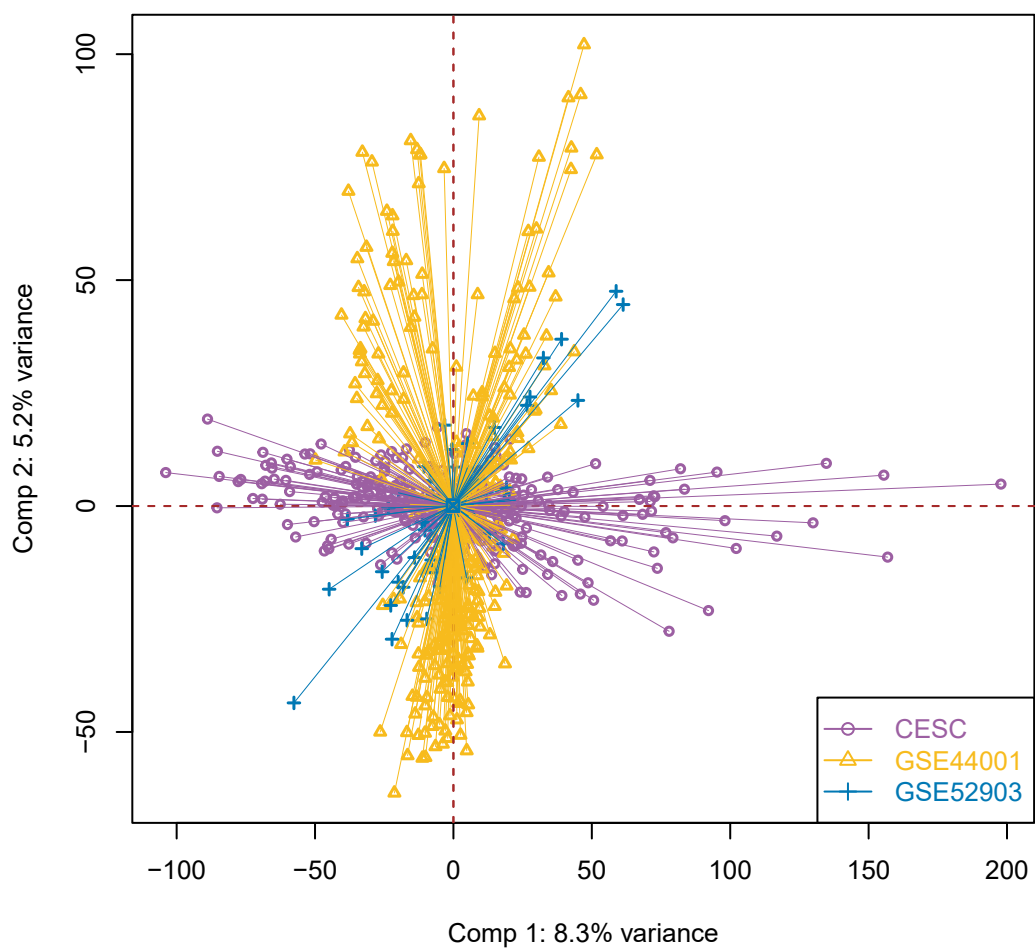

Supplement: Supplementary file 1 [file Presentation1.PDF]
